# Vaccine Procurement: A Conceptual Framework Based on Literature Review

**DOI:** 10.3390/vaccines9121434

**Published:** 2021-12-03

**Authors:** Vincenza Gianfredi, Antonietta Filia, Maria Cristina Rota, Roberto Croci, Lorenzo Bellini, Anna Odone, Carlo Signorelli

**Affiliations:** 1 School of Medicine, Vita-Salute San Raffaele University, Via Olgettina 58, 20132 Milan, Italy; croci.roberto@hsr.it (R.C.); bellini.lorenzo@hsr.it (L.B.); signorelli.carlo@hsr.it (C.S.); 2 Department of Infectious Diseases, Italian National Health Institute, Viale Regina Elena Rome 299, 00161 Rome, Italy; antonietta.filia@iss.it (A.F.); mariacristina.rota@iss.it (M.C.R.); 3 Department of Public Health, Experimental and Forensic Medicine, University of Pavia, 27100 Pavia, Italy; anna.odone@unipv.it

**Keywords:** vaccine procurement, Europe, review, vaccine, immunization

## Abstract

Ensuring timely access to affordable vaccines has been acknowledged as a global public health priority, as also recently testified by the debate sparked during the COVID-19 pandemic. Effective vaccine procurement strategies are essential to reach this goal. Nevertheless, this is still a neglected research topic. A narrative literature review on vaccine procurement was conducted, by retrieving articles from four academic databases (PubMed/MEDLINE, Scopus, Embase, WebOfScience), ‘grey’ literature reports, and institutional websites. The aim was to clarify key concepts and definitions relating to vaccine procurement, describe main vaccine procurement methods, and identify knowledge gaps and future perspectives. A theoretical conceptual framework was developed of the key factors involved in vaccine procurement, which include quality and safety of the product, forecasting and budgeting, procurement legislation, financial sustainability, and plurality of manufacture, contracting, investment in training, storage and service delivery, monitoring and evaluation. This information can be useful to support policymakers during planning, implementation, and evaluation of regional and national vaccine procurement strategies and policies.

## 1. Introduction

Vaccines are considered one of the most important medical achievements that save and improve the lives of millions of people globally each year. However, in order to be beneficial, national immunization programmes require uninterrupted availability of high quality and affordable vaccines, as highlighted by the World Health Assembly [1]. State-of-the-art technologies are available to supervise vaccine development and implement security checks in most parts of the world. However, more attention should be brought to vaccine procurement mechanisms. 

Vaccine procurement is a complex process composed of a pre-buying phase (identify needs, select prequalified products and suppliers, establish specifications and legal criteria, prepare for bidding and evaluation) and a post-buying phase (ensuring availability, timeliness, monitoring the safety, and reporting performance). This cycle involves continuous interplay between public health professionals, national policymakers, international regulators (e.g., WHO, EU), and manufacturers [1]. Procurement mechanisms are meant to reach objectives of equity, accessibility, and high vaccination coverage [2,3].

In the current European Union (EU) legislative framework, National Immunization Programs (NIPs) and individual Member States (MSs) have to assess needs and take care of supply and forecast potential vaccine shortages, which can have detrimental public health effects. Moreover, a reflection on vaccine procurement is more than ever necessary, especially in light of the COVID-19 pandemic. Indeed, a well prepared and integrated vaccine procurement will be highly beneficial both to ensure a timely and appropriate storage of vaccines, and to avoid inequalities among countries.

The aim of the current review is to clarify key concepts about vaccine procurement, and to describe the intricate vaccine procurement system. The final aim of this review was to develop a theoretical conceptual framework of vaccine procurement useful in deeply understanding the complexity behind, and to provide a supportive tool for those professionals involved in, the vaccine procurement process.

## 2. Materials and Methods

### 2.1. Research Design

A narrative literature review of vaccine procurement was conducted in March 2020. The process was documented and the results were processed following the SANRA (Scale for the Assessment of Non-systematic Review Articles) guidelines [4].

Both academic and grey literature were included. The following scholarly databases were screened for peer-reviewed articles: PubMed/Medline, EMBASE, WebOfScience, and Scopus. The grey literature examined included documents such as public reports, archival records, policy briefs, and various studies and books on vaccine procurement. Additional sources used were government websites (i.e., of national health authorities) and websites of the following institutions: World Health Organization (WHO), WHO Regional Office for Europe, European Centre for Disease Prevention and Control (ECDC), Vaccine European New Integrated Collaboration Effort (VENICE) Project, European Council, and the European Commission (EC). 

Sources were searched using a combination of the following keywords: “supply”, “procurement”, “supply and distribution”, “strategic stockpile”, and “vaccines”, “immunization programs”, “vaccination”, pooling text word terms, and MeSH, according to the searched database.

### 2.2. Inclusion and Exclusion Criteria

The title and abstract of retrieved references were screened to assess for consistency with the aims and the topic of the search. References highlighting the characteristics and impact of procurement strategies were presented. We only included references in English and with full text available. In order to perform a comprehensive analysis, reducing the possibility of losing studies, no time filter was added.

### 2.3. Data Extraction and Reporting

Full texts were downloaded only for references that met inclusion/exclusion criteria based on title and abstract. Only articles that reported characteristics and impact of procurement strategies were included. Data extraction was conducted only for these articles, using a pre-defined and pre-piloted spreadsheet in Microsoft Excel^®^ for Windows (Redmond, WA, USA, 2007). Qualitative data recorded included the following: name of the first author, year of publication, country(ies) where the study was conducted, publication type, study period, study topic/aim, type of vaccine studied, and main results. Publication type was categorized according to PubMed/National Library of Medicine’s 2020 MeSH terms [5]. References were managed using the citation manager software EndNote^®^ X 4.0.2 (Clarivate Analytics, Australia). The content analysis aimed to describe a wide range of factors implicated in the complex vaccine procurement system and identify lack of knowledge and future perspective. The study findings were presented using thematic content analysis, structuring the narrative literature around the themes derived from the included studies.

## 3. Results

### 3.1. Conceptual Framework for Vaccine Procurement

**Figure 1:**
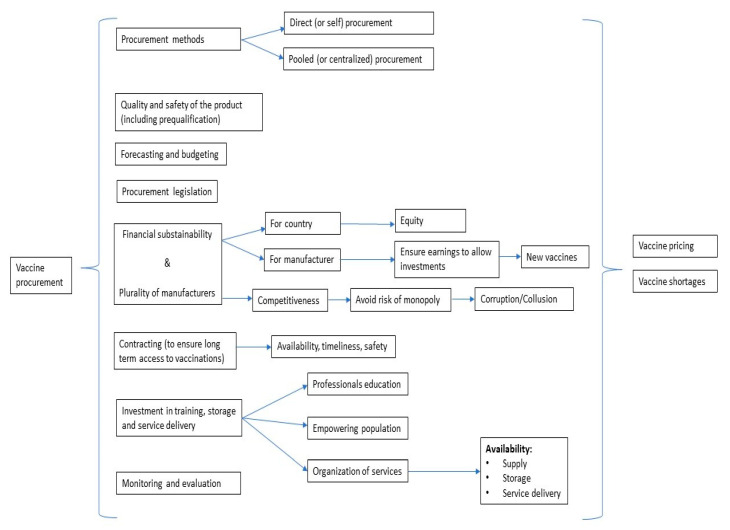
Flow diagram of the selection process.

Based on the information retrieved from the literature review (included studies and main characteristics are reported in Supplementary Table S1) a theoretical conceptual framework of vaccine procurement, depicted in Figure 1, was developed, aiming at describing the main interplaying factors in the procurement process. Vaccine procurement is a multidimensional process in which several and interconnected aspects play essential roles. The main aspects to consider in vaccine procurement are the following: procurement methods (centralized and self-procurement), quality of the products (including vaccine safety and prequalification), planning needs (forecasting and budgeting), procurement legislation, financial sustainability (for both countries and manufacturers, including plurality of manufacturers), transparency of vaccine pricing, contracting (including market research capacity, market intelligence, and access to price data that is as current as possible), high-value based investments (training, storage, service delivery), and monitoring and evaluation. This cycle involves continuous interplay between public health professionals, national policymakers, international regulators (e.g., WHO, EU), and manufacturers [6]. Each of the above points will be discussed below.

### 3.2. Relevant Definitions of Procurement Methods and Purchase Mechanisms

The World Health Organization identifies two main strategies (or methods) of vaccine procurement [7]:

- Direct (or self) procurement: countries are autonomous in their decisions and do not have to comply with WHO prequalification requisites. Three different main purchase mechanisms are used, depending on the country size, needs/shortages, and bargaining power: competitive bidding, request for quotation, and sole-source procurement. Competitive bidding is a “procurement process in which clearly stated product specifications and contract requirements are issued to multiple suppliers to solicit pricing and performance responses”; request for quotation is a process where “offers (quotations) are requested from several prospective suppliers without employing formal sealed bidding procedures”; sole source procurement refers to “purchasing from a single manufacturer without competition among potential suppliers”.
- Pooled (or centralized) procurement: refers to a mechanism by which several countries (buyers) combine into a single entity that purchases vaccines on their behalf. The WHO has identified four levels of pooled procurement [8]: in level 1 (information sharing and individual informed buying), procurement is conducted by each country, but information about suppliers and products is shared. Benchmarks and best practices are identified. In level 2 (coordinated informed buying), procurement is conducted by each country. Market research is jointly performed. Supplier performance and prices are monitored via shared information, which may also involve the national political level. In level 3 (group contracting), procurement is conducted individually. Still, vaccines are prequalified, prices set, suppliers selected, and products purchased by the process of joint actions and negotiations. In this context, legal framework, procedures, and policies need to be completely harmonized. Group contracting is a useful example of economies of scale. Lastly, in level 4 (central contracting), tendering, awarding contracts and delivery are coordinated by a single representative organization, which can also implement supplementary technical and functional roles. Centralized procurement is traditionally performed within supranational entities (e.g., PAHO) [9], while decentralized procurement “occurs when administrative responsibility, authority, and discretion are delegated to service and sub-service delivery personnel” [10].

Procurement methods are a crucial aspect of immunization since they can have a relevant impact on guaranteeing a proper and sustainable vaccine supply. In most cases, the bottleneck for vaccine supply is the lack of a well-structured supply chain capable of facing logistic problems [11,12]. In addition, due to the complexity of vaccine production, any sudden unanticipated changes in vaccine needs may result in shortages [13]. Therefore, this should also be taken into account in order to avoid shortages due to ineffective needs assessments that ignore extraordinary circumstances.

### 3.3. Quality and Safety of Vaccines (including Prequalification)

“Vaccines of assured quality” are defined by WHO as products that “consistently meet(s) appropriate levels of purity, potency, safety and efficacy as judged through an independent review system competent to take an evidence-based decision” [14]. All the above-mentioned aspects are assessed before licensure of a vaccine by national regulatory authorities. However, post-licensure surveillance is also critical to monitor the effectiveness and ongoing safety of new vaccines [15,16]. Indeed, vaccines are perishable and temperature-sensitive products that need careful management in terms of preparation, packaging, transportation, storage, and administration with high-quality controls throughout the whole chain.

Countries’ national regulatory authorities (NRA) play an important role in ensuring the quality of vaccines procured. The WHO has identified the need for countries to have an independent NRA that can perform six critical functions: licensing; post-marketing surveillance; lot releasing, laboratory access for testing (in order to respond to reported adverse events); good manufacturing practice inspections; clinical evaluation [17].

Prequalification by the WHO is a mechanism that seeks to ensure that vaccines used in immunization programmes meet global standards of quality, safety, and efficacy [18]. It is a service provided by WHO to United Nations (UN) and other procurement agencies to aid them in making purchasing decisions and is considered an interim solution for countries that are yet to develop national capacity for procurement and regulatory processes. WHO’s role is strictly interlaced with that of NRAs, as vaccines need to be licensed by NRAs prior to availability [19,20]. Unlike other drugs, vaccines are subject to lot release procedures. Given the limited number of manufacturers and the high barriers to market entry, disregarding prequalification processes can unleash durable reverberations in terms of shortages and price increase [18]. In spite of this emergency, in recent years, some major European countries have experienced a trend towards disinvestment in primary prevention, including vaccination procurement [21]. Indeed, even if the healthcare expenditure increased, vaccine spending reduced markedly, especially in Germany, Spain, and France [21]. Reasons explaining this decline are not clear. Some hypotheses are the reduction in vaccination coverage, the changes in the market competitive landscape, and changes that occurred in the vaccination schedules [21].

Vaccine quality also depends on providers, medical and administrative personnel, who must avoid storage and handling errors. Healthcare providers have a pivotal role as the last keepers of the entire safe vaccine handling process [22].

### 3.4. Forecasting and Budgeting

Assessing the needed quantity of vaccines is an essential first step within the procurement process [23]. Forecasting demands is essential to provide sufficient quantity of vaccines to avoid shortages and program disruptions [24], in most of the countries performed by the National Immunization Technical Advisory Group (NITAG) [25]. If a country’s forecasts are not accurate, this will impact procurement decisions, for example inadequate forecasting will lead to more emergency procurement [17].

When planning vaccine needs. The risk of a surge in vaccination demand among the public should also be taken into account. An example is provided by the yellow fever outbreak which put a severe strain on vaccine supplies in Angola during 2016 [26]. As recently argued by a Global Alliance for Vaccines and Immunisation (GAVI) market analysis [27], preparedness implies meticulous stockpiling and capacity building. In the EU context, a report of the EU-Joint Action on Vaccinations, highlighted the need to adopt a cross-national approach to assess the predictability of vaccine supply and the suitability of current stockpiling, especially for vaccines against epidemics and emerging infectious diseases [28]. The authors suggest a European-wide data repository as a viable instrument to share data and information [28].

Regarding budgeting, the resources required for a NIP are determined, either within the annual budget for the Ministry of Health (MoH) (with a specific budget line-item for vaccine procurement), or within a multi-year budgeting aligned with immunization program planning.

### 3.5. Procurement Legislation

A national drug policy provides a legislative and regulatory base from which appropriate decisions about the procurement characteristics of health sector goods can be made [29]. Legal provisions for vaccination include language in laws, regulations, ministerial or executive decrees, and other legal documents requiring the State, healthcare providers, or patients to take action to increase the population’s uptake of vaccines.

Specifically, rules on procurement could allow flexibility to conduct multi-annual tenders, providing open tender process (such as in Lithuania, Macedonia, and Romania) or directly negotiating with companies (such as in Croatia) [30]. Moreover, characteristics of vaccine procurement rules might also attract tenders, by promoting a cross-border procurement. Such characteristics include delegation of authority, drafting of language documentation, electronic communication, and other requirements. Indeed, the bid, as well as all correspondence and documents relating to the bid exchanged by the bidder and the purchaser (from the confirmation of bidder’s qualifications and eligibility to participate in tender to all labeling and packaging inserts) shall be written in the language specified in the Bid Data Sheet (special conditions of contract and technical specifications for each purchase). Moreover, purchasers are encouraged to provide as many documents as possible in revisable, electronic format. This will expedite bid preparation, reduce the number of inadvertent mistakes made by bidders, and, as a result, simplify the evaluation. Electronic communication is also important for sellers because they must inform the buyer by fax or telex many days in advance of each shipment of goods [29].

Procurement legislation could also include flexibility in joint procurement that would allow countries the option to self-procure some products. In these cases, countries are not required to wait for WHO recommendations or prequalification, although most rely on WHO guidance and prequalification before implementing a new vaccine. Self-procurement allows countries to customize procedures to meet their own individual needs, laws, rules, and regulations [7]. Legislation should also foresee possible implications of judicial rulings which may influence public vaccine tenders [31].

Lastly, a National Regulatory Authority is necessary to oversee and monitor imports and locally produced products. Such an authority, which is often an autonomous part of a national drug regulation body, must be properly staffed and have the authority to license vaccines for use in the country, to establish procedures for lot release and to create a post-marketing surveillance structure. It is crucial also for establishing appropriate safety stocks [29].

### 3.6. Financial Sustainability for Both, Countries and Manufacturers (including Plurality of Manufacturers and Domestic Production)

The vaccine market is very fragile, mainly because vaccines are biological products with a relatively short shelf-life and potentially high risk of contamination. Financially, their market features high start-up and fixed production costs and a pool of risk-averse institutional buyers, which causes relatively unpredictable returns in terms of final price per unit [17]. The high costs are mainly due to expensive investments in technology, processes, and oversight [32]. However, developers’ costs are not restricted to the production phase of currently available vaccines. Instead, they are amplified by R&D expenditures required for meeting competitive challenges and developing new vaccines [17]. In this perspective, the vaccine market is not easy to access for start-ups or smaller private sector actors. Nevertheless, low vaccine price is one of the measures used to estimate the effectiveness of purchase mechanisms. Indeed, low vaccine price per unit might enable countries to buy sufficient vaccine supply, thus guaranteeing equitable access to the population, and help finance high-value based investments on the National Immunization Program (NIP).

Overall, vaccine price needs to be equally sustainable for both countries and manufacturers [32]. Indeed, if, from the countries’ point of view, prices should be affordable enough to ensure the system’s economic sustainability, manufacturers need adequate compensation to guarantee uninterrupted production, vaccine security, and timely delivery [17]. This equilibrium is fundamental to ensure a plurality of suppliers, with stable and reliable production capacity, and to support sufficient innovation. Unrewarding vaccine prices might disincentivize manufacturers, causing them to reduce appropriate investments to ensure reliable supply and new vaccines [33].

Vaccine manufacturers plurality is instrumental in maintaining market competition, which, in return, results in higher quality standards, as it fuels constant research to offer better products on the market [34]. Furthermore, it can also generate higher value services associated with vaccine purchase. Price-based procurement methods tend to favor bigger manufacturers, who can afford to offer their products at lower prices. These procurement methods, especially if followed by lengthy contracts with a limited number of winners, usually exclude smaller manufacturers from the market [13]. Indeed, these would-be competitors lack the financial power to withstand market risk dynamics, and, if faced with no investment returns given their exclusion from contracts, they usually leave the market. Consequently, less competition paves the way to monopolies, increased risk of shortages, potential corruption, and an increase in vaccine prices, quite the contrary to the intended result [13].

A limited number of manufacturers may also diminish the bargaining power of public procurers [35]. In order to prevent this issue, in 2016, GAVI proposed multiple winners’ tenders as a potential solution [33]. In the specific case of vaccine procurement, market risk-sharing can be defined as the buyer’s will to participate in the investment made by the manufacturers to develop new products and enter the market. Market risk-sharing could let smaller companies compete with larger corporations by reducing financial damage and maintain competition in case of failure to win the tender [33,36]. Other proposed strategies involve direct loans to small manufacturers, subsidies from private donors, or milestone payments for achieving specific results (e.g., clinical or regulatory). This last mechanism, in particular, is a form of development cost-sharing.

Usually, new vaccines are more expensive immediately after being introduced into the market. Their price generally decreases over time, following license expiration, as new manufactures are allowed to enter the market and widen the supply to meet the increased demand built over time. The overall economic effect results in lower vaccine prices according to the demand and supply law [37]. However, for new vaccines, we might also consider that new combined formulations reduce the number of inoculations and vaccine wastage and lower the vaccine costs in the long run. Vaccine cost is somehow a broader concept than vaccine price, as it incorporates the combination of price plus all the costs of supply, storage, administration, staff wages, and vaccine campaigns [33].

The WHO, through its Market Information for Access to Vaccines (MI^4^A) report, highlighted the paucity of manufacturers in the market as a core challenge to vaccine supply assurance [38]. This can result in high prices and significant vulnerability to production problems threatening the vaccine supply [39]. GAVI suggested creating a manufacturers’ portfolio to assure multi-manufacturer tendering (a concept defined as “bundling”), and to create a roadmap to shape the market and promote cooperation between members, in order to guarantee supply safety and long-term competition between manufacturers [33,40].

According to the World Bank, and as suggested by GAVI [33], domestic production might be a solution to supply problems, especially for low- or middle-income countries where immunization programs may substantially differ from those of high-income countries [17]. However, domestic production may be useful in high-income countries too. For instance, since the Eurozone is a large recipient and source of biotech companies’ investment, Wilsdon and colleagues envisage an opportunity to further strengthen economic ties and development in vaccine production [13,41]. The EU receives roughly 71% of all vaccine R&D investments, it produces over 80% of all global doses, and it hosts 45% of overall producers [42]. Apart from economic aspects, strong domestic vaccine industry may prove an invaluable asset to face shortages and other health emergencies [34]. The 2009 H1N1 pandemic provides an instructive example. As the epidemic was predicted to become uncontainable, demand for the vaccine reached an all-time high worldwide, leading to vaccine shortages [34]. In similar circumstances, depending solely on international providers may threaten the safety of vaccine supply. A working industry still has the ability and the tools to eventually reconvert its production to face health emergencies, like specific vaccine manufacture. Such an option is not available if the domestic industry is absent.

### 3.7. Contracting

Contracting refers to the ability to contract long-term purchases in order to ensure the production of vaccines without interruption or unpredictability in the supply. In light of this, ensuring a long-term vaccine supply is the main aim of a well-structured contracting. However, many aspects might affect the contracting performance. Firstly, a country (ies) should be equipped with a well-prepared market intelligence able to improve market research including access to price data that is as current as possible; as well as have available adequate capacity, knowledge, and processes in place for preparing bidding documents. According to WHO, increasing the transparency of vaccine prices reduces the existing information asymmetry between vendee and vendor, helping to set prices in a more adequate, fair, and efficient way [37]. Vaccine-pricing transparency is also beneficial in monitoring the vaccine market over time and to evaluate which of the several factors involved might impact more on vaccine price.

Higher transparency of vaccine pricing would provide valuable information to policymakers and would help in better-informed decision-making on both vaccine procurement and new vaccine introduction strategies [30,43]. Moreover, according to Hinsch et al., higher vaccine pricing transparency positively impacts on the quality of products, negotiations, national pricing policies, and on reducing the prices [44].

The WHO/UNICEF Joint Reporting Process collected performance data about several components of immunization systems, including vaccine prices [45]. According to the latest report, vaccine pricing information is, in most cases, available at national level, although the relatively low response rate limits the generalizability of this finding (overall response rate = 43%, with lower participation among upper-middle and high-income countries) [37]. Nevertheless, these data are rarely made publicly available and accessible [37]. In this perspective, in 2001, the World Health Assembly passed Resolution 54.11, outlining the need for improving monitoring of drug pricing for more equitable drug access [46], followed by a new resolution, in 2019, urging to publicly share prices information, mainly through web-based tools [47]. However, despite many efforts, also performed by WHO with the V3P project (later mentioned), little progress has been made in increasing vaccine-pricing transparency, and new regulatory strategies should be put in place to achieve better price monitoring. In fact, even if several countries have some legal obligations to publish vaccine prices, most of the time they are not regularly and timely updated, and often data cannot be disaggregated to perform comparisons [48].

WHO’s Vaccine Product, Price and Procurement (V3P) is a project aimed at increasing transparency to make vaccine prices comparable and to provide accurate information to countries [8]. Based on the results obtained by the V3P project, WHO built the MI4A/V3P database collecting data on vaccine prices, volumes, manufacturers, procurement modalities, and contract lengths [39]. The amount of such information has been increasing yearly, reaching the highest rate in 2019 [39]. Nevertheless, merely comparing the vaccine price among countries cannot represent a benchmark [49], since vaccine prices are highly dependent on several factors, such as product details (for instance, type, formulation, and presentation), procurement context, the volume of purchase, and country (income, taxes, and legislations) [39].

According to Dimitri et al., increasing transparency can also produce undesired consequences [50]. More specifically, it may lead to a higher risk of collusion if only a few local competitors are selected as potential providers (may not be applicable to all countries). Indeed, excessive price transparency may be used to enforce cartel dynamics, in which a group of manufacturers manipulate prices by punishing potential transgressors of pre-arranged deals between cartel members [51].

### 3.8. Investment in Training, Storage and Service Delivery 

In the last decade, new vaccines have been developed, mainly against meningitis B, pneumococcus, rotavirus, and Human Papilloma Virus (HPV), and more recently, COVID-19 vaccines, increasing volumes of vaccines that needed to be packed and shipped. New products might have had a strain on the capacity and vaccine supply and logistics. Additional investments have been required, including professional updating, immunization promotion campaigns, new cold chain tools, launching supply chain revitalization efforts, and equipment renovations [52].

Training health personnel and informing the general population are two sides of the same coin. On the one hand, healthcare workers must be kept confident and up-to-date about vaccine novelty, effectiveness, and safety. On the other hand, such efforts are vain if the public is ill informed about the delivery mode and beneficial effects of vaccinations [53]. In fact, according to the 2019 European Commission report [54], lack of training of healthcare workers and lack of immunization information registers are the two most frequent barriers to high VC. Missing or incomplete vaccine schedules cause loss of investments, both due to the underutilization and disposal of existing vaccine stocks and to the cost of health assistance of unvaccinated subject which can become ill (or to contain the spread of epidemics). Therefore, promoting informative and educational campaigns is a high-value based investment.

Logistics should also be considered for the product to reach the target population. A significant example is provided by Mozambique, a low-income country where supply bottlenecks impaired effective vaccine distribution, and VC were sub-optimal [11]. Following the implementation of modeling procedures and the involvement of the Ministry of Health and other parties in educational workshops, the supply chain was vastly improved [11]. In essence, VC increased while the cost of vaccine administration per capita was reduced, sometimes even by roughly 30% of the original cost [11].

### 3.9. Monitoring and Evaluation

Evaluating and monitoring the effectiveness of vaccine procurement strategies requires a well-defined set of indicators [29]. According to the most authoritative health institutions, the vaccine market can be defined as healthy if it is able to guarantee a stable vaccine production over time, in sufficient quantities, safe in terms of effects on health, diversified in the offer, and sustainable in costs. GAVI suggested considering indicators able to monitor VC, supply, shortages, price, and innovation [33]. Among process indicators, the operational performance is the most important aspect and should be referred to the prioritization of interventions such as planning needs and assessing the impact of strategies on VC and procurement outcomes. Notably, according to Nelson et al., close monitoring of vaccine procurement outcomes could provide an advocacy tool to drive down vaccine prices and costs [55]. Evaluations should be conducted before, during, and after an implementation strategy to identify limits and strengths and potential good practices. Identified indicators should be part of periodic reports published and available because, in this context, transparency at each level is highly recommended.

### 3.10. Vaccine Shortages

The literature review highlights that vaccine shortages occur frequently, mainly in middle income countries but also in high income countries. These can lead to missed vaccinations and higher occurrence of vaccine-preventable diseases, some of which may be deadly [56]. For example, a recent European survey found that 19 of 21 participating countries, reported at least one stockout or shortage event between 2016 and 2019 [56]. Vaccine shortages have been registered in many countries worldwide. In the USA, shortages have been registered for hepatitis A (Hep A) and Herpes Zoster Virus vaccines [57], both linked to higher demand. Japan suffered vaccine shortages due to a natural disaster occurring in the area where the domestic vaccine-producing factory was located; this episode highlighted the necessity to build vaccine stockpiles [58]. It also experienced a flu vaccine shortage in 2003 due to increased demand following an outbreak in specific regions, which was partially solved through targeted redistribution of vaccine supplies [59]. New Zealand suffered a BCG vaccine shortage, which was determined by a global shortage from 2015 to 2018 [60]. Canada faced a Hep B vaccine shortage from 2017 to 2019 due to increased demand [61], particularly from developing countries. It also experienced Hep A and rabies vaccine shortages [62]. Australia has reported several vaccine shortages, two of which are ongoing, an inactivated influenza vaccine shortage linked to seasonal depletion of stock and an oral typhoid vaccine shortage due to changes in commercial viability [63].

None of the above published articles assessed whether vaccine shortages were linked to the procurement method used. The European survey also investigated type of procurement methods used by participating countries and found that this is performed mainly at national level by the public sector [56]. Higher numbers of shortage/stockout events were reported by the one country that reported procuring vaccines exclusively at subnational level and by one country that procures vaccines through the private sector, but it was not possible to establish a correlation between procurement level and number of shortages [56]. The authors concluded that future research could better investigate the relationship between procurement mechanism and resilience to shortages. Shortages were addressed by purchasing additional doses, stockpile consumption, or reallocation [56]. In conclusion, vaccine supply should be carefully planned to take into account all the above-mentioned features in order to avoid shortages due to ineffective needs assessments that ignore extraordinary circumstances [13].

## 4. Discussion

This article presents a theoretical conceptual framework for vaccine procurement, based on a literature review on this topic. According to the results of our review, to build an appropriate vaccine procurement system, several aspects need to be considered.

As for vaccine quality, prequalification is an essential means of vetting which has implications on safe vaccine selection, shortages prevention, and price control. Vaccine pricing is strictly related to high cost involved in production, research, and development. For this reason, final price per unit should take into account both country’s need to guaranteeing equitable, timely, and uninterrupted access to the population, and manufacturers’ adequate compensation. The balance between the two interests is crucial to ensure a plurality of suppliers, stable production capacity, and to avoid risk of monopoly and corruption/collusion. Undoubtedly, a country’s ability in planning needs is fundamental in order to guarantee an accurate preparedness, to prevent and avoid shortage, as well as vaccine wastage. An example of how important it is to plan the needs, can be retrieved from Italy during the flu vaccine campaign during the 2020/2021 season. Since differential diagnosis between COVID-19 and influenza or influenza-like illnesses might be complicated by their similar clinical presentation, with frequent symptom overlapping, the Italian Ministry of Health issued a Circular recommending to expand the target population that should receive the influenza vaccine free-of-charge during the pandemic [64]. However, since flu vaccine procurement must be planned well in advance (February-March) some Italian regions experienced difficulties in purchasing enough doses to meet the new population’s needs. 

Alternative vaccine plans, like adjusting vaccine schedules and using different formulations, coupled with stakeholders’ involvement, can be of help in facing extraordinary needs. In the EU, a platform devised to share information about shortages among MS has been proposed [56]. GAVI, in the Supply Procurement GAVI’s report, suggested creating a manufacturers’ portfolio to assure multi-manufacturer tendering (a concept defined as “bundling”), and to create a roadmap to shape the market and promote cooperation between members, in order to guarantee supply safety and long-term competition between manufacturers [33,40].

Moreover, appropriate planning needs might ultimately affect vaccine price, since due to the economies of scale effect, cost per unit decreases with increasing scale. It is clear that all of these aspects (vaccines’ safety, availability, accessibility, and price) directly and indirectly affect vaccine coverage [65,66]. For this reason, within the vaccine procurement process, high-value based investments, including campaigns for empowering people, for training the health care workers, and for innovating information technology infrastructures should be considered. 

Lastly, in order to ensure the healthiness of the vaccine procurement system, a periodic evaluation and monitoring of the applied vaccine procurement strategies is needed. In this perspective, a set of well-defined indicators should be used. Among them, vaccine price and transparency, vaccine coverage, shortage episodes, supply, and level of innovation are potential indicators suggested by GAVI. 

### Limits and Strengths 

The current study has several limitations. First of all, the available literature on vaccine procurement is scarce and mostly limited to low-middle income countries. This greatly reduces the generalizability of the findings and their possible application to high-income countries. Moreover, retrieving original primary studies was difficult, as this required verifying numerous sources ranging from academic databases to government websites. Additionally, entry and indexed terms were not homogeneous. Most of the documents consulted were grey literature and reports from international agencies.

Notwithstanding all its potential limitations, the current review could represent a reference for those who are interested in the topic, scholars, policymakers alike, and public health experts. 

## 5. Conclusions

The results of our review show that choices performed within the vaccine procurement process (both at national and supranational level) might have important consequences on the vaccine ecosystem and can indirectly affect public health [67]. Although the study was limited by the fragmentation of the available literature, it represents an attempt to comprehensively discuss and present the different aspects of vaccine procurement. The results accrued allow to state that vaccine procurement is an intricate process and several aspects such as vaccine safety, quality of the product, costs, needs and supply, as well as transparency, competitiveness, and high-value based investments, should be considered. All these aspects, if well balanced, concur synergistically in building sustainable vaccine procurement systems able to prevent vaccine shortage risk and lower costs, increasing overall accessibility and equity. Secondly, at the European level, a reflection on vaccine procurement is necessary, even more so in the present emergency context of the COVID-19 pandemic. Indeed, immediately after the announcement from the pharmaceutical companies of new safe and effective vaccine availability, several high-income countries expressed their will to start an exclusive financial relationship with the pharmaceutical companies. Obviously, this reaction immediately increased the alleged preemption risk for the candidate vaccine. In this context, the risk of jeopardizing world population health was extremely high as well as the increment of health inequalities. In light of this, the European Commission strongly promoted a central COVID-19 vaccination procurement, making available large amounts of vaccine stocks with a relatively low price, and consequently guaranteeing an affordable access to all the European countries. However, despite the high value of this initiative, one of the most contested aspects was the several vaccine shortages registered especially at the beginning of the mass vaccination campaign. Protestors mainly accused the EU commission of low transparency and those contracts mainly pointed to achieving the lowest vaccine price, instead of guaranteeing compliance with the contractual terms by the manufacturers. This event is clearly related to the maintenance of the financial sustainability of the vaccine market. Indeed, it is imperative to think beyond the simple price and comprehensively evaluate the costs that take into account all processes related to logistic management and vaccine production.

The recent experience of the SARS-CoV-2 pandemic has clearly shown how having a well-planned vaccine procurement system is a priority to guarantee the vaccination offer and a social and economic sustainability at national level.

To conclude, it would be useful to conduct an ex-post analysis of the ongoing central procurement for COVID-19 vaccines and encourage future research in this area, particularly to estimate benefits and costs, supply, and storage, for both self and pooled procurement.

## Data Availability

All the data supporting reported results can be found either in the manuscript or the enclosed supplementary materials.

## Supplementary Materials

The following are available online at www.mdpi.com/article/10.3390/vaccines9121434/s1, Table S1: Descriptive characteristics and main results of the retrieved publications included in the review (in alphabetical order).
