# Supplementary material for: Vaccine Procurement: A Conceptual Framework Based on Literature Review
Source: Vaccines (Basel). 2021 Dec 3;9(12):1434. doi: 10.3390/vaccines9121434 (PMC8707219; doi:10.3390/vaccines9121434)
Supplement: Supplementary file 1 [file vaccines-09-01434-s001.zip › Supplementary Table S1.pdf]

vaccines

MDPI

Table S1. Descriptive characteristics and main results of the retrieved publications included in the review (in alphabetical order).

Reference [Ref]	Year	Country/ies	Publication type	Study period	Topic/Aim	Vaccine/s	Main results
**Peer-reviewed articles**							
Coyne [20]	2019	N/A (worldwide)	Journal article (Editorial)	1974-2018 (historical perspective)	Historical perspective on WHO PQ program for vaccines, medicines and medical devices	N/A	As of 2019, 148 vaccines are WHO-prequalified.
Dellepiane [18]	2015	N/A (worldwide)	Journal article (Review/Historical Article)	1987-2012	Historical perspective and future of WHO vaccine PQ program, including capacity building programs	All	As of 2015, 127 vaccines are WHO-prequalified. Over the years, increased role, funding and statutory requirements for candidate vaccines in most fields: technical evaluations, inspections, site visits and audits. From mid-1990s, WHO started implementing capacity building programs to ensure NRAs of LICs are fully functional.
DeRoeck [29]	2003	PAHO EPI Revolving Fund, Gulf Cooperation Council, Croatia, Macedonia, Lithuania and Romania	Technical Report	N/A	Group Procurement and harmonization of practices	BCG, DTP, Hib, IPV, MMR, OPV, TT	Pooled procurement requires nations to sign cooperation agreements and increase transparency to tackle differences that may impair effective group procurement.
Duclos [25]	2013	188 WHO countries	Journal article	2012	Analyzes the 2013 WHO/UNICEF Joint Reporting Form of NITAGs results	N/A	The majority of countries (n = 92, 52%) report NITAGs with appropriate legal basis. Procurement is among the areas of expertise of core NITAG members.

Ethgen [21]	2016	7 Western European Countries (Germany, France, Italy, Spain, Sweden, Portugal, UK - England only)	Journal article (original research)	2008-2014 (main dataset)	Analyzing and comparing the evolution of national-level public expenditure on vaccines (proportion of national healthcare spending)	All national vaccination calendars	All countries but Sweden (+5.9%) saw a reduction in public expenditure on vaccine procurement (Italy -9.6%, Spain -6.7%, Germany -6.2%, France -4.2%)
Hinsch [42]	2014	GAVI Graduating countries and Middle Income Countries	Journal article (original research)	2011-2013	Vaccine Price Transparency and its advantages	N/A	Accurate data play an important role in increasing price transparency. Data and information provided may also contribute to price decreases and other market effects.
Huff-Rousselle [3]	2012	Worldwide	Journal article (Review)	1996-2009	Benefits of pooled procurement in the pharmaceutical goods market.	N/A	Cooperation between countries in Pooled Procurement results in lower prices, decreases corruption and costs and increases quality and equity.
Kartoglu [16]	2014	Worldwide	Journal article (Review)	N/A	Compares and contrasts thermostability of vaccines, identifies technical and regulatory solutions to improving cold chains	27 vaccines, 7 "traditional" (e.g. YF, OPV, DTP) and 20 "new" (e.g. HPV, RV, JE)	Identified solutions:<br>1) Technical: helping companies select more stable candidate vaccines, using VVMs, recognize and prevent freezing, improving temperature control, removing ice in favor of cool water packs for in-country transport, training professionals.<br>2) Regulatory: harmonize, implement and enforce national and supranational guidelines within the vaccine procurement process
Lee [11]	2016	Mozambique	Journal article (Review)	Unclear (2014-2016)	Describes strategies to improve vaccine supply chain in Mozambique	All	Improving procurement logistics by modeling tools and on-field interventions resulted in increased vaccine availability (~+ 10-30% in the 2 provinces) and price per dose fell (~ 0.1 US$).

Lydon [12]	2017	WHO and UNICEF Member States	Journal article	2010-2015	Vaccine stockouts around the world	BCG, DTP containing vaccine, Measles containing vaccine, Polio	More than one-fifth (23%) of stockout events reported to WHO/UNICEF JRFs are due to national-level procurement delays.
Malhame [38]	2019	All 73 GAVI-supported countries	Journal article (Review/Historical Article)	2005-2020 (projected)	Describes market-shaping actions for a GAVI-sponsored pentavalent vaccine	Pentavalent DTP-HepB-Hib	Phases of GAVI's vaccine procurement policies 1) 2005-2010: technical support to NRA, demand forecasting 2) 2011/2015: risk-sharing agreements, long-duration tenders 3) 2016-2020: more focused pricing strategy, new type of tender (rapid price decrease). Results: over the study period, price per dose drop from ~3.5$ to <1$, annual demand rose from ~ 50 to ~300 million-doses (mainly fueled by India).
McCue [10]	2000	N/A (theory),	Journal article (Review)	2000 (survey)	Describes current trends in public procurement (all items, not restricted to vaccines)	N/A	Outlines theoretical models of centralized, decentralized and mixed public purchasing systems. Untangles the decision-making processes, focusing on efficiency, control, and accountability levels.
Nelson [53]	2014	The 23 Development Assistance Committee (DAC) countries	Journal article (original research)	2009-2011	Vaccine procurement outcome monitoring	N/A	Monitoring of vaccine procurement outcomes may prove to be a useful tool to adjust vaccine costs.
Pagliusi [34]	2019	Developing Countries Vaccine Manufacturers Network	Conference Proceeding	2019	Sharing of best practices, access to vaccines and regulatory convergence	All	Successful partnerships lead to product innovation, accelerated vaccine prequalification and inclusion into NIPs, as well as better access.

Rey-Jurado [32]	2018	Worldwide	Journal article (Review)	N/A	Overview of domestic manufacturers	All	India, Brazil: State-owned national manufacturers provide almost all vaccines. Germany: 90% of vaccines purchased from private sector. USA: public and private producers coexist.
Shamsi [23]	2018	All (focus on the US)	Book chapter	N/A (focus on 2004-2006 flu seasons)	Procurement challenges in supply management	All (special focus on influenza)	Proposed solutions to procurement challenges: Sourcing: interconnecting supply chains. Avoiding shortages: increase R&D investments, advance purchase agreements, public/private partnership. Purchase: relying on multi-sourcing (flu seasons 2004-2006).
Stuurman [33]	2018	Denmark, Finland, Netherlands, Norway, Slovenia, Italy, Sweden, Belgium, France, Germany, Greece, Spain, UK	Abstract	2017	European Procurement Systems and vaccine brand diversity	N/A	Vaccine brand diversity is dependent upon the vaccine procurement system. Sub-National procurement systems result in higher brand diversity.
Thompson [24]	2016	Worldwide	Journal Article (Review)	2014	Hypothesizes optimal global stockpiling strategies	'Universal' (measles-containing) vs. 'non-universal' (oral cholera)	Explores dynamics of global vaccine supply and demand to consider opportunities to develop and maintain optimal global vaccine stockpiles for universal vaccines, characterized by large global demand (for which we use measles vaccines as an example), and nonuniversal (including new and niche) vaccines (for which we use oral cholera vaccine as an example)

							Authors conclude that global health policy leaders and stakeholders should procure and maintain appropriate global vaccine rotating stocks for measles and rubella vaccine now to support current regional elimination goals, and should probably also do so for other vaccines to help prevent and control endemic or epidemic diseases.
Wilsdon [13]	2020	Germany, Italy, Spain and Romania	Journal article (Review)		Theoretical comparison of price-based tenders vs innovative strategies	Measles-containing, influenza and hexavalent DTaP-Hib-IPV-HepB	Price-based tenders have disadvantages: they could obstacle R&D investments, cause shortages and do not contribute directly to increasing coverage rates. More flexible and functional procurement models should be based on value-added services.
Zaffran [51]	2013	Worldwide	Journal article (Review)	2000-2013	Current issues and a global plan of action to strengthen supply chain and logistics.	All	Availability: procurers should consider 'cost per dose delivered', rather than 'cost per dose procured'. Streamlining and integrating supply chains is also vital.
Zerhouni [62]	2019	Worldwide	Journal article (Review)	2001-2019	Historical perspective on the birth and role of the GAVI alliance	N/A (All GAVI-supported vaccines)	GAVI-sponsored procurement resulted in a 21% increase in immunization coverage in LICs (2000-2018). 73 supported countries and 15 transitioned out of GAVI support as of 2018.
**"Grey literature" (governmental reports, technical reports, deliverables …)**							
Dimitri [49]	2006	Worldwide	Handbook	N/A	Procurement features	N/A	Among other findings, increasing transparency in procurement practices may result in more frequent collusion practices.
EU-JAV (Filia et al.) [54]	2019	18 EU-JAV countries + 3 EU/EEA non-EU-JAV Countries	EU-JAV Report – Work Package 6 – Task 6.1	January 2016 – February 2019	Survey on vaccine procurement in 21 EU/EEA countries, both EU-JAV and non-Members, assessing	N/A (all)	Procurement mainly performed by public sector (87%) at national-level. Most frequent shortages: DT-containing vaccines most of the time at national level (89.4%). Causes: quality issues and global shortage = 60%. Solutions: supply chain

		(Estonia, Hungary, Ireland)			shortages/stockouts episodes, and exploring causes and possible solutions		improvements, and centralize (European) stockpiling, joint procurement (even if difficult to implement).
EU-JAV (Johansen et al.) [27]	2019	21 EU-JAV Countries	EU-JAV Report – Work Package 6 – Task 6.2	2019	I. Improving forecasting, supply and stock. II. Concept analysis for a EU data repository on vaccine market features	N/A (all)	I. Suggested mechanisms: warning systems to prevent stockouts, emergency stockpiling, comprehensive national-level overview of demand and stocks, government agencies help in meeting future NIP changes.<br>II. Only 4/20 countries agree that a EU data repository would prevent shortages
GAVI [30]	2002	All GAVI-supported countries	Technical report	2000-2002	Market analysis, stakeholder perspective, review of GAVI 2000-2001 procurement activities	N/A	- Market analysis: rise in R&D investments ($750 M in 2000)<br>- Procurement review: overall, only 18% of awarded vaccine doses were purchased<br>- Challenges: time pressure, ineffective partnerships
GAVI [31]	2016	All GAVI-supported countries	Technical report	2011-2020 (forecasted)	Reviews the 2011-2015 period, proposes future procurement strategies	All	2011-2015 review: market-shaping objectives mostly achieved (supply, cost)<br>Strategic priorities for 2016-2020: 'healthy market' framework, long-term view on investments and markets, support to country-owned decisions.
PAHO (WHO Regional Office) [9]	2011	WHO Region of the Americas (35 Member States)	Technical report	N/A	PAHO Revolving Fund's purchase procedures for vaccines (and other equipment)	All	Procedures: yearly forecasting, quality standards via WHO (or international entities) prequalification, all phases of procurement are RF supervised. Special financial agreements are possible for States requiring more vaccines than the allotted funds.
World Bank (Bumpas, J.) [17]	2008	4 L-MICs (Bangladesh, Botswana,	Technical report	Nov 2007- May 2008	Comparative review of vaccine procurement practices and	All	Main country-specific issues:<br>- Bangladesh: transparency, delays<br>- Botswana: delays, quality, planning needs

		Nigeria, Pakistan) enjoying World-Bank financed vaccine procurement			recommendations to strengthen NRAs		- Nigeria: availability (local distribution and logistics)<br>- Pakistan: quality, legal specifications, lack of health system capacity)
World Health Organization (IVB Department) [28]	2003	Worldwide	Book	N/A	Reference manual for vaccine public procurement	All	Describes procurement options, methods, procedures, specifications, quality assurance and the then-existing supranational procurement entities (UNICEF, World Bank)
World Health Organization [48]	2007	Worldwide	Congress	N/A	Principles underlying regional pooled procurement.	N/A	Key Enablers: Shared Political Will, collecting relevant international or regional benchmark prices, sharing of information and experiences.
World Health Organization [46]	2015	Worldwide	Electronic Supplementary Materials	2015	Vaccine price transparency	N/A	Improving vaccine price transparency by publicly sharing information is not easy despite legal obligations.
World Health Organization (IVB Department) [6]	2018	Worldwide	Webpage	N/A	Defines vaccine procurement mechanisms and systems	All	Direct procurement: CB, RFQ, SSP<br>Pooled procurement: 4 levels<br>Outsourcing to agencies: UNICEF, PAHO RF
World Health Organization [45]	2019	Worldwide	Congress	2019	Vaccine market transparency	All	In order to increase vaccine market transparency, WHO urges to publicly share price information, particularly using web-based tools.

World Health Organization [47]	2019	Worldwide	Electronic Supplementary Materials	2019	Factors influencing vaccine prices	N/A	Comparing vaccine prices between different countries must be adjusted for all the logistic and background differences.

Abbreviations: AEFI = adverse events following immunizations. CB = competitive bidding. CMYP = comprehensive multi-year planning for immunization. DT = diphtheria and tetanus vaccine. DTP = diphtheria, tetanus and pertussis vaccine. EEA = European Economic Area. EMRO = Regional Office for the Eastern Mediterranean. EU = European Union. GAVI = Global Alliance for Vaccines and Immunization. HIC(s) = high-income countrie(s). IVB = Immunization, Vaccines and Biologicals. JRF = Joint Reporting Form. HepB = Hepatitis B. Hib = Haemophilus influenzae type b. ICG = International Coordinating Group. IPV = inactivated poliovirus vaccine. LICs = Low-Income Countries. L-MICs = Low-Middle Income Countries. NITAGs = National Immunization Technical Advisory Groups. NIPs = national immunization program(s). NRAs = National Regulatory Authority. N/A = not applicable. OCV = oral cholera vaccine. PAHO RF = Pan American Health Organization Revolving Fund. PMS = post-marketing surveillance. PQ = prequalification. R&D = research and development. RFQ = request for quotation. SSP = sole-source procurement. UNICEF = United Nations Children's Fund. US = United States of America. VVMs = vaccine vial monitors. YF = yellow fever vaccine.
